# Beam Damage Detection Under a Moving Load Using Random Decrement Technique and Savitzky–Golay Filter

**DOI:** 10.3390/s20010243

**Published:** 2019-12-31

**Authors:** Hadi Kordestani, Chunwei Zhang, Mahdi Shadabfar

**Affiliations:** 1 Structural Vibration Group, Qingdao University of Technology, Qingdao 266033, China; hadi@qut.edu.cn; 2 Department of Geotechnical Engineering, Tongji University, Shanghai 200092, China; mahdishadabfar@tongji.edu.cn

**Keywords:** random decrement technique, Savitzky–Golay filter, time-domain analysis, damage detection, moving sprung mass, baseline free

## Abstract

In this paper, a two-stage time-domain output-only damage detection method is proposed with a new energy-based damage index. In the first stage, the random decrement technique (RDT) is employed to calculate the random decrement signatures (RDSs) from the acceleration responses of a simply supported beam subjected to a moving load. The RDSs are then filtered using the Savitzky–Golay filter (SGF) in the second stage. Next, the filtered RDSs are processed by the proposed energy-based damage index to locate and quantify the intensity of the possible damage. Finally, by fitting a Gaussian curve to the damage index resulted from the non-damage conditions, the whole process is systematically implemented as a baseline-free method. The proposed method is numerically verified using a simply supported beam under moving sprung mass with different velocities and damage scenarios. The results show that the proposed method can accurately estimate the damage location/quantification from the acceleration data without any prior knowledge of either input load or damage characteristics. Additionally, the proposed method is neither sensitive to noise nor velocity variation, which makes it ideal when obtaining a constant velocity is difficult.

## 1. Introduction

Generally, the input excitations and output responses are needed for accurate identification of modal parameters. However, in some cases, such as heavy traffic load on bridges, the input excitations can hardly be measured. In such cases, where the operational loads exerted on the structure are not available, the output-only-based methods can be used as a proper workaround, which have received great attention in structural health monitoring (SHM) [1,2]. It has been proved that vibration data (e.g., acceleration data) have some damage signatures that cannot be observed by the naked eye [1]. Therefore, signal processing is necessary to provide a visible form of these signatures. Some of these signal processing-based methods are transformers [3,4,5,6,7,8], time-domain methods (e.g., random decrement technique (RDT)) [1,2,9,10,11,12,13,14], and source separation methods (e.g., blind source separation (BSS)) [15,16,17,18,19]. To implement a systematic damage detection procedure, a nested loop of RDT and a Savitzky–Golay filter (SGF) is employed in the present paper.

Pakrashi experimentally identified the crack evolution in bridge-type structures under moving load utilizing the distortion in wavelet coefficients of the strain signal [3]. Hester and Gonzalez [4], Balafas and Kiremidjian [5], and Cantero and Basu [6] employed wavelet transformers to analyze the acceleration data and visualize these singularities. Although they proved that the wavelet transform is a great tool in bridge health monitoring (BHM), the sensitivity of wavelet analysis in noisy environments could not be neglected [7]. In addition to noise sensitivity, the road irregularities, velocity variation, having small velocity, and wheel dimension were other issues that limit the application of wavelet analysis [8].

RDT is a promising output-only time-domain approach that employs a special averaging technique to calculate the random decrement signature (RDS). The aim of this technique is to average the transient response of the signal out. Lee et al. [11] employed the RDT to find the free response of a bridge under traffic load. He et al. [12] developed a method using a combination of RDT and empirical mode decomposition to identify the modal parameters of a bridge under moving loads. Wu et al. [13] combined the RDT with mode separation techniques and determined the modal parameters of cables in a cable bridge. A well-documented example of a systematic procedure for the design and implementation of SHM using RDT is addressed by Buff et al. [14]. Kordestani et al. [2] experimentally verified that RDSs have the signatures of the damage in a tied-arch bridge under moving load.

BSS is an output-only source separation approach that aims to find the input loads from output responses. BSS utilizes different techniques such as second-order blind identification (SOBI) to determine the modal parameters of structures during the source separation procedure. Loh et al. [19] employed SOBI to locate the damage in a bridge structure and compared it with other damage detection methods. Huang and Nagarajaiah [18] combined SOBI with wavelet analysis, improved the performance of SOBI, and successfully found more modal parameters of the bridge with limited sensors.

To the knowledge of the authors, only few studies addressed the direct use of time domain filters in health monitoring and damage detection. Among them, the application of a moving average filter (MAF) in noise reduction and damage localization can be mentioned. Since the moving average filter (MAF) has the ability of noise reduction, some researchers only highlighted the use of MAF to enhance the accuracy of the BHM system [20,21,22]. Direct use of MAF in BHM was also addressed in a few studies in which the damage was numerically and experimentally located along the bridge type structures [1,23]. It should be noted that the MAF is the zero-order SGF and the SGF is a time-domain smoothing filter that finds a trend line for a curve using an nth-order polynomial function in its kernel [24,25]. Some researchers demonstrated the experimental application of SGF on the modal curvature to detect the damage in the plate-like structure [26,27]. The application of SGF on enhancing the results of SHM using a neural network algorithm was also addressed and experimentally verified by means of an alloy plate [28].

The problem of having slow constant velocity was mentioned in many of the above literature. Additionally, noise sensitivity and baseline were other issues that limited vibration-based damage detection methods. Moreover, recording displacement in bridge structures is not easy; therefore, using acceleration data received much attention in the field of BHM. Accelerometers can be easily used and installed virtually at any location on the structure to provide a wide range of sampling frequencies. Because of the low cost and small size of accelerometers, they can significantly reduce the cost of BHM.

**Figure 1:**
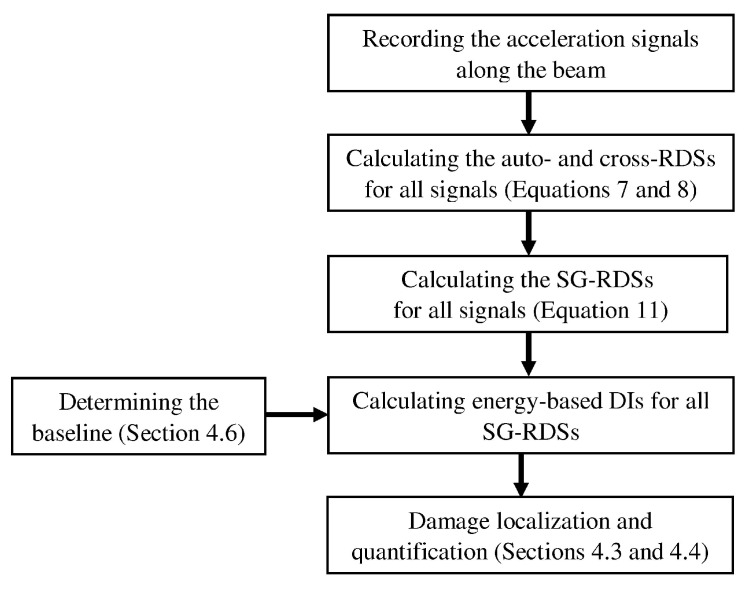
General flowchart of the proposed damage detection method.

The aim of this paper is to develop a vibration-based damage detection method utilizing SGF that does not have the above problems. This paper employs SGF to calculate a special trend line for each RDS (hereafter referred to as Savitzsky–Golay RDS (SG-RDS)). Therefore, this paper proposes a two-stage time-domain output-only damage detection method where the damage is localized based on the energy-based damage index (DI) calculated from SG-RDSs. Each SG-RDS shows a special energy content. The structural damage changes the pattern of energy distribution along the beam. Consequently, the energy-based DI locates and quantifies the damage using the change imposed in the pattern. To verify the proposed method, a simply supported beam was numerically modeled under moving sprung mass and a set of acceleration responses were recorded along the beam. The results showed that the proposed method is able to accurately locate and quantify the damage using an energy-based DI calculated from SG-RDSs. Moreover, a Gaussian curve was used to simulate the baseline and formulate the damage detection process as a baseline-free method. The overall view of the proposed method is schematically drawn in Figure 1.

## 2. Basic Theories and Methodology

### 2.1. Theory of RDT for a Single-Channel Signal

Introduced by Cole in 1968 [29], the RDT was developed as a promising output-only time-domain approach that employs a special averaging technique to calculate the (RDS). Since the aim of this technique is to average the transient response of the signal out, the equation of motion can be rewritten using RDS with zero input excitation [2]. There is no general mathematical basis available for RDT. In this regard, considering four limitations for input excitations (i.e., stationary, random white noise, zero-mean, and Gaussian), Vandiver et al. [30] provided a strict form of the mathematical basis for RDT. Under such limitations, the RDS well behaves as a free vibration.

The strict mathematical basic provided for RDT is illustrated in [2,30]. Assuming a single-channel time history signal obtained from a linear system, the equation of motion for such a system is expressed as:

(1) $$M\ddot{X}\left(t\right)+C\dot{X}\left(t\right)+KX\left(t\right)=f\left(t\right),$$

where M, C, and K indicate mass, damping, and stiffness matrices, respectively, and X(t) represents the displacement time history recorded in the vertical direction. Vandiver et al. [30] supposed that the *f*(*t*) is random white noise, zero-mean, Gaussian, and stationary input excitation. Assuming that Equation (1) is satisfied at any time instant ((e.g., $t_{i}$), a simple shift in time (τ) leads to:

(2) $$M\ddot{X}\left(t_{i}+\tau \right)+C\dot{X}\left(t_{i}+\tau \right)+KX\left(t_{i}+\tau \right)=f\left(t_{i}+\tau \right).$$

Considering the input excitation to be zero-mean, the mean value of $N_{R}\left(N_{R}\to \infty \right)$ for different signal instants $\left(t_{i}\right)$ averages the transient response out of the signal. Mathematically speaking:

(3) $$\frac{M}{N_{R}}\sum_{i=1}^{N}\ddot{X}\left(t_{i}+\tau \right)+\frac{C}{N_{R}}\sum_{i=1}^{N}\dot{X}\left(t_{i}+\tau \right)+\frac{K}{N_{R}}\sum_{i=1}^{N}X\left(t_{i}+\tau \right)=\frac{1}{N_{R}}\sum_{i=1}^{N}f\left(t_{i}+\tau \right).$$

Defining γ(τ) values, as shown in Equation (4), Equation (3) are converted into a free vibration form as expressed in Equation (5).

(4) $$\gamma \left(\tau \right)=\frac{1}{N_{R}}\sum_{i=1}^{N}X\left(t_{i}+\tau \right),$$

(5) $$M\ddot{\gamma }\left(\tau \right)+C\dot{\gamma }\left(\tau \right)+K\gamma \left(\tau \right)=0.$$

**Figure 2:**
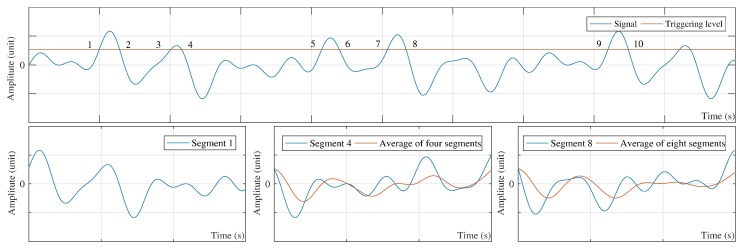
The basic concept of special averaging in random decrement technique (RDT).

In the literature, γ(τ) is known as RDS [9,10,14,31,32]. The method for selecting the $N_{R}$ of different instants is to make RDT a special averaging technique by means of a so-called triggering condition. The triggering condition cuts off the signal at $N_{R}$ different times and provides $N_{R}$ segments with the same amplitude at the first of each segment (Figure 2). Next, RDS is calculated using the average of the resulting segments. Some recommended values for the triggering condition addressed in the literature are listed in Table 1 [9,32,33].

The level-crossing triggering condition is utilized in this paper because of its simplicity. Although *a* is an arbitrary value in the level-crossing triggering condition, a good recommendation can be made assuming $a=\sqrt{2}\times$ standard deviation [2,9]. Therefore, Equation (4) can be re-written as:

(6) $$\gamma \left(\tau \right)=\frac{1}{N_{R}}\sum_{i=1}^{N}X\left(t_{i}+\tau \right)|X\left(t_{i}\right)=a.$$

Equation (6) is the exact mathematical form of Figure 2 if the level-crossing triggering condition is considered. In Equation (6), $N_{R}$ and τ are the number of segments and the length of each segment, respectively. The level-crossing triggering condition, *a*, controls the number of $N_{R}$. Therefore, taking the value of *a* close to the average of the signal leads to the highest number of $N_{R}$ and significantly increases the time and cost of computation. Although the minimum acceptable value for $N_{R}$ is 100, the stable result is achieved at 1000 [34]. For the parameter τ, the resulted RDS should represent a full frequency content of the original signal [2].

### 2.2. Theory of RDT for Multi-Channel Signals

**Figure 3:**
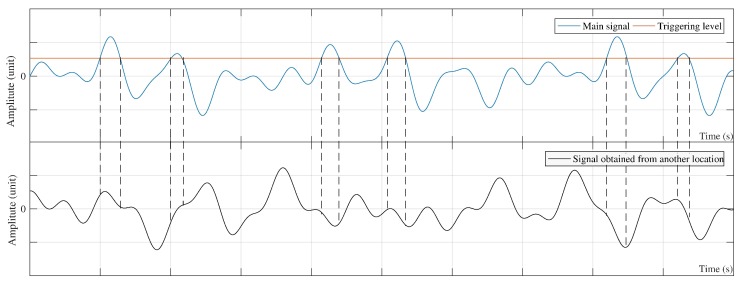
Concept of cross-random decrement signature (RDS) calculation.

All the concepts formulated above are related to a single-channel signal. In some cases, a set of sensors are required to be installed along with the structure such as a bridge. Therefore, RDT should be applied to multi-channel signals. To this end, two techniques can be adopted. The first one is to consider each signal separately and then apply the RDT to the signal. The second is to select one signal as the main signal and calculate the RDSs of the remaining signals according to the main signal. The first approach is fundamentally similar to the single-channel signal. To implement the second technique, a signal should be first set as the main signal with its corresponding RDS known as auto-RDS [32]. The triggering information of the auto-RDS can then be used to calculate the RDSs of other signals referred to as cross-RDSs. This can be mathematically formulated as:

(7) $$\gamma {}_{m}\left(\tau \right)=\frac{1}{N_{R}}\sum_{i=1}^{N}X{}_{m}\left(t_{i}+\tau \right)|X{}_{m}\left(t_{i}\right)=a,$$

(8) $$\gamma {}_{c}\left(\tau \right)=\frac{1}{N_{R}}\sum_{i=1}^{N}X{}_{c}\left(t_{i}+\tau \right)|X{}_{m}\left(t_{i}\right)=a,$$

where $\gamma {}_{m}\left(\tau \right)$ and $\gamma {}_{c}\left(\tau \right)$ are the auto- and cross-RDS, respectively. In Equations (7) and (8), the indices *m* and *c* denote the main and cross signals, respectively. It should be noted that the triggering information is the same for Equations (7) and (8). Figure 3 shows the concept of Equations (7) and (8).

The process of calculating the RDSs sets the initial value of auto-RDS equal to the level-crossing triggering condition, while the initial value of each cross-RDS is equal to the average value of its segments. Therefore, the noise of auto-RDS is less than that of cross-RDSs [2,10]

### 2.3. Theory of SGF for a Signal

The SGF employs the least-square fit to approximate a higher-order polynomial impulse, smooth the signal, and reduce the noises [24,25,35,36]. Therefore, the filter kernel of SGF is a polynomial approximation of an impulse. To smooth the signal, SGF calculates a special polynomial trend line for the value at time $t_{i}$ using the neighboring values. Next, it substitutes the actual value at time $t_{i}$ with the approximated one resulted from the trend line. To further explain the formulation of the SGF, suppose there are 2S+1 consecutive data as $y_{t_{i}-S}$,$y_{t_{i}-S+1}$,*…*,$y_{t_{i}}$,*…*,$y_{t_{i}+S}$ from a signal $y_{t}$ (all the data around time $t_{i}$). 2S+1 is the number of observations used to calculate the trend line of the signal using SGF, hereafter referred to as SGF span. Here, the time $t_{i}$ is in the middle of the SGF span. Hence, the polynomial function, F(τ), can be considered as follows:

(9) $$F\left(\tau \right)=\sum_{i=0}^{i=r}\beta_{i}\tau^{i}=\beta_{0}+\beta_{1}\tau +\ldots +\beta_{r}\tau^{r},$$

where *r* and $\beta {}_{i}$ are the order and coefficients of the polynomial function, respectively. The least-square fit using this function requires minimizing the following equation:

(10) $$T\left(t\right)=Min\left\{\sum_{\tau =-S}^{\tau =S}{\left(y_{t+\tau }-F\left(\tau \right)\right)}^{2}\right\},$$

where the T(t) represents the trend line. Then, the signal is smoothed by substituting $y_{t}$ with T(t). Therefore, an individual trend line is required for each signal observation. To use Equation (10), the order and span of SGF should be determined in advance. Once the order of SGF is chosen, the coefficients of Equation (9) (i.e., $\beta {}_{i}$) will be numerically calculated using Equation (10). De Oliveira [28] utilized a SGF with order = 10 and span = 501 to implement their SHM system. Using the SGF in civil engineering, especially in SHM field, is a relatively new topic. Unfortunately, there is no particular recommendation available in the SHM literature for choosing the order and span of SGF. In this paper, for SGF with order = 3, the authors recommend a formula to determine the SGF span according to the first natural frequency as: SGF span = (sampling frequency)/ (first natural frequency). For example, if the sampling frequency is 2000 Hz and the first natural frequency of the signal is 2 Hz, the best SGF span is the closest odd number larger than 2000/2=1000 (i.e., 1001) observations. To calculate the SG-RDS, Equation (10) is rewritten as follows:

(11) $$SG-RDS\left(t\right)=Min\left\{\sum_{\tau =-S}^{\tau =S}{\left(RDS_{t+\tau }-F\left(\tau \right)\right)}^{2}\right\}.$$

## 3. The Numerical Model of a Simply Supported Beam Subjected to Moving Sprung Mass

**Figure 4:**
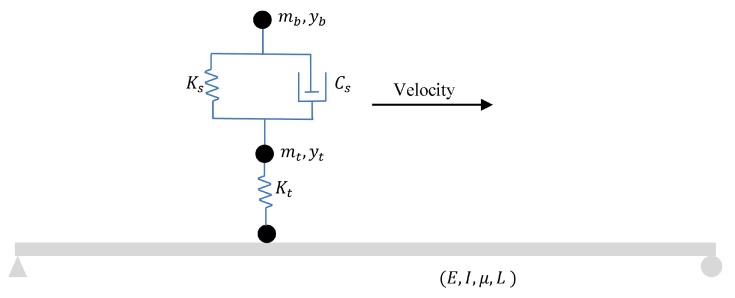
The schematic view of the simply supported beam under moving sprung mass.

The damage detection of a simply supported beam under different moving loads has received great attention over the past few decades [1,3,20,21,23,37,38]. Truckloads are generally modeled by three different methods: (1) Moving force, (2) moving mass, and (3) moving sprung mass. Ouyang [39] mathematically proved that the moving force excites a wide range of frequency and the resulting vibration is similar to a vibration force. Additionally, the bridge natural frequency shifts due to the interaction of modal parameters of the vehicle and bridge–vehicle [40,41,42]. Since the moving force has no mass and spring, it cannot be regarded as an appropriate moving load problem. In this section, a moving sprung mass with a constant velocity and an Euler–Beroulli beam are used as a numerical model of a simply supported beam under a quarter car [23,43,44]. A schematic view of the model is shown in Figure 4. The details of the numerical model are given in Table 2 and Table 3. The rotational degree of freedom of the sprung moving mass was constrained so that it could only vibrate vertically while moving along the simply supported beam with constant speed. The natural frequency of this simply supported beam in the non-damage condition without moving load is 2.93 Hz. Considering the sampling frequency of 2000 Hz, the SGF order and span in non-damage condition are 3 and 683, respectively.

**Figure 5:**
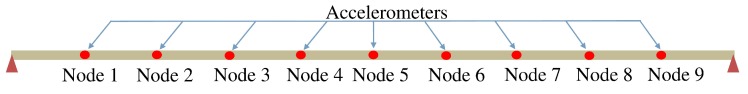
Schematic location of nodes to obtain acceleration data.

To examine the influence of speed variation, the moving sprung mass passed over the simply supported beam with three different velocities (i.e., 1.25, 2.5, and 4 m/s) listed in Table 3. With each pass of moving sprung mass, the acceleration signals were recorded at nine nodes along the simply supported beam with a fixed distance of 2.5 m and the sampling frequency of 2000 Hz. The schematic location of these nine nodes is shown in Figure 5. In this numerical model, five different damage scenarios were considered as listed in Table 4. Damage can be introduced to the beam using different approaches such as changing the stiffness or mass distribution. In this paper, however, the damage was modeled by reducing the section area in order to simulate the crack at the bottom of the beam. The ratio of damage is also determined by dividing the crack depth to the beam height ratio.

## 4. Results and Discussion

### 4.1. Acceleration Signals, RDSs and SG-RDSs

**Figure 6:**
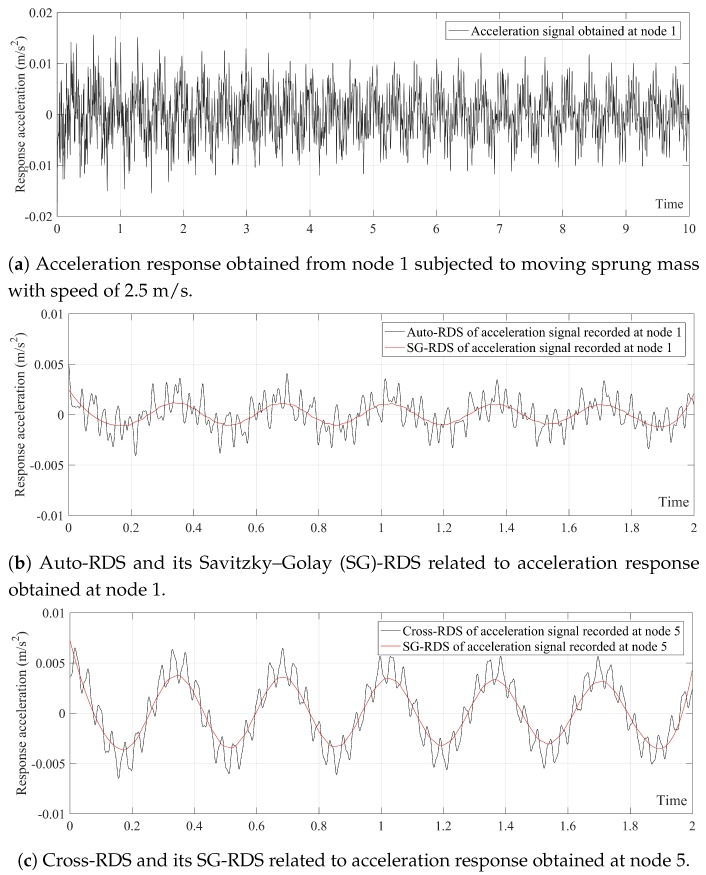
(**a**) Acceleration response recorded at node 1 subjected to moving sprung mass with speed of 2.5 m/s. (**b**,**c**) are the auto- and cross-RDSs with their corresponding SG-RDSs.

There are six different conditions for the simply supported beam (i.e., five damage scenarios + one non-damaged condition). Considering nine nodes along the bridge, there would be 6 × 9 = 54 raw acceleration signals for the simulation in each velocity. Given three different velocities in each scenario, 54 × 3 = 162 raw acceleration signal are finally resulted. Providing all the raw acceleration signals in the paper might be difficult. As such, Figure 6a presents the acceleration signal obtained from node 1 subjected to the moving sprung mass at velocity 2.5 m/s as an example. The other cases are provided in a suplimentary Excel file along with paper containing all the raw acceleration signals obtained from the numerical simulation. These acceleration signals are used to calculate the corresponding SG-RDSs based on Equation (11). Since each numerical model has a different standard deviation for its main signal, a suitable level-crossing triggering condition should be less than $a=\sqrt{2}\times$ standard deviation for all main signals. In this study, the level-crossing triggering condition (i.e., a=0.005 m/s${}^{2}$) is considered for all models. The signal obtained from node 1 in each numerical model is regarded as the main signal. Figure 6b,c depict the auto-and cross-RDSs and their corresponding SG-RDSs. The length of each RDS is considered to be 2 s.

According to Figure 6b,c, SG-RDSs show only one natural frequency, which is exactly the natural frequency of the simply supported beam. At both edges of the signal, SG-RDS has a larger amplitude, because there are not enough data around the points near the two edges. This problem can be easily solved by shortening the SG-RDSs. Hence, only 1 s of SG-RDSs (from 0.5 to 1.5 s) was used.

### 4.2. Energy-Based DI

Choosing modal parameters was common practice to develop a DI, monitor the modal behavior, and determine the possible damages (if there is any) [2]. It has been proved that the DIs that are based on natural frequency (as the most common modal parameter) are not sensitive enough to detect the local damages [20,45,46]. Therefore, researchers have paid more attention to non-modal parameters to develop new forms of DI [1,2,47,48]. Kordestani et al. [1,2] employed energy-based DI to locate the damage position. The energy-based damage index provides three main advantages. First, it is sensitive to the damage signatures in the signal. Second, it can locate the damage with no need to determine the modal parameters. This DI reduces the time and cost compared to modal parameter-based DI. Third, it can be implemented by a simple integration algorithm. The SG-RDS energy can be calculated as:

(12) $$E_{SG-RDS}=\int {\left(SG-RDS\right)}^{2}dt,$$

where $E_{SG-RDS}$ is the energy of SG-RDS. $E_{SG-RDS}$ should be calculated for all SG-RDSs. In the following, a normalization factor $\mu_{i}$ is considered for this purpose:

(13) $$\mu_{i}={\left(\frac{E_{{SG-RDS}_{i}}}{\bar{E_{SG-RDS}}}\right)}_{non-damage\;condition},$$

where $\bar{E_{SG-RDS}}$ is the average of SG-RDS energy for each node. $E_{{SG-RDS}_{i}}$ is the energy of SG-RDS at node *i*. The parameter $\mu_{i}$ is an influential term that is further discussed in Section 4.6. This parameter should be calculated for all nodes only in non-damage condition. Finally, having obtained the parameter $\mu_{i}$, the new energy-based DI is calculated as:

(14) $${DI}_{i}=\frac{100}{\mu_{i}}\times {\left(\frac{E_{{SG-RDS}_{i}}}{\bar{E_{SG-RDS}}}\right)}_{damage\;condition}.$$

Equation (14) normalizes all DIs to 100. The damage in the simply supported beam changes the energy distribution along the beam so that the DIs close to the damage location show values more than 100.

### 4.3. Damage Localization of the Simply Supported Beam

**Figure 7:**
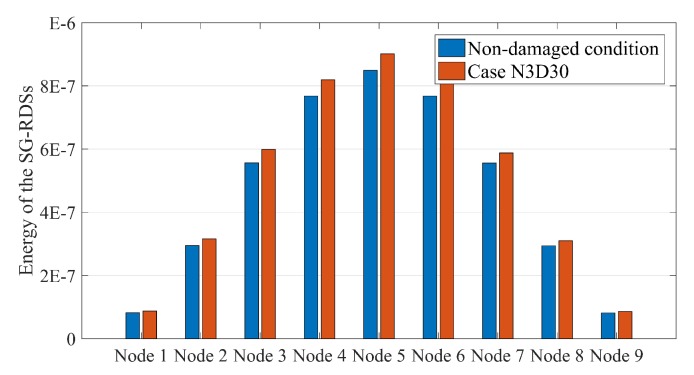
Distribution pattern of energy of SG-RDSs along a simply supported beam subjected to moving sprung mass with speed 2.5 m/s. The blue bars are a non-damaged condition. The orange bars are the damaged condition for case N3D30.

Figure 7 depicts the energy of SG-RDSs under the moving sprung mass with a speed of 2.5 m/s in the two cases of non-damaged condition and N3D30. As can be seen, the energy distribution of SG-RDSs follows a Gaussian pattern along the beam in the non-damaged condition, and the occurrence of damage slightly influences the pattern.

**Figure 8:**
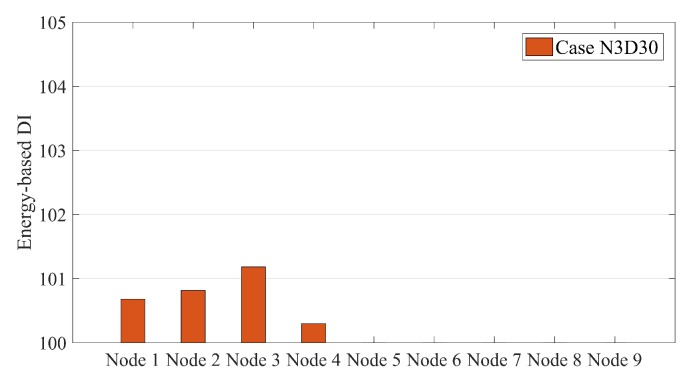
Energy-based damage indices (DIs) along a simply supported beam subjected to moving sprung mass with a speed of 2.5 m/s for case N3D30.

Figure 7 exhibits that SG-RDSs have more energy in the damaged condition. However, the location of the damage cannot be determined using this Figure. Once the normalization parameter ($\mu_{i}$) is calculated for the non-damaged condition, the energy-based DI converts Figure 7 into Figure 8. Figure 8 shows that the energy-based DI is at a maximum at the damage location. Since the energy-based DI is 100 for all nodes under the non-damage condition, the vertical axis of Figure 8 begins at 100. This makes the damage more visible in the bar-chart form.

**Figure 9:**
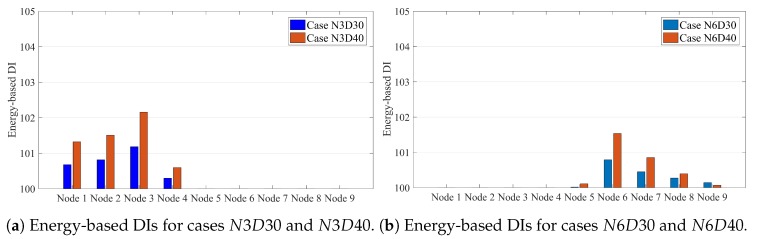
Energy-based DIs along a simply supported beam subjected to moving sprung mass with a speed of 2.5 m/s for (**a**) Cases N3D30 and N3D40 and (**b**) Cases N6D30 and N6D40.

**Figure 10:**
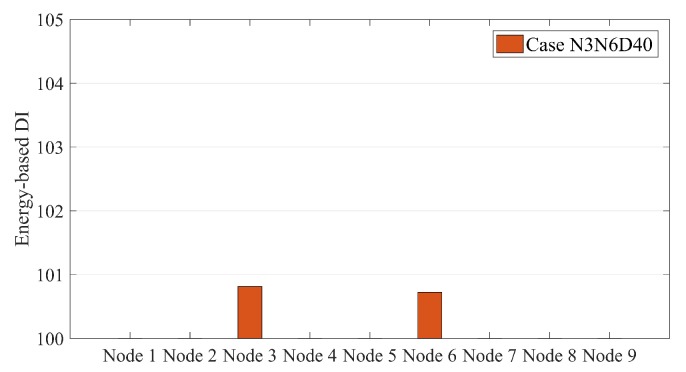
Energy-based DIs along a simply supported beam subjected to moving sprung mass with a speed of 2.5 m/s for multi-damage case N3N6D40.

Figure 9 shows the energy-based DIs for all single damage scenarios under moving sprung mass with a speed of 2.5 m/s. Figure 10 presents the multi-damage scenario where two damages occurred at nodes 3 and 6.

Figure 9 and Figure 10 illustrate that the energy-based DI can accurately locate the damage and present the maximum value at the damage locations.

### 4.4. Damage Quantification of the Simply Supported Beam for Single Damage Scenarios

**Figure 11:**
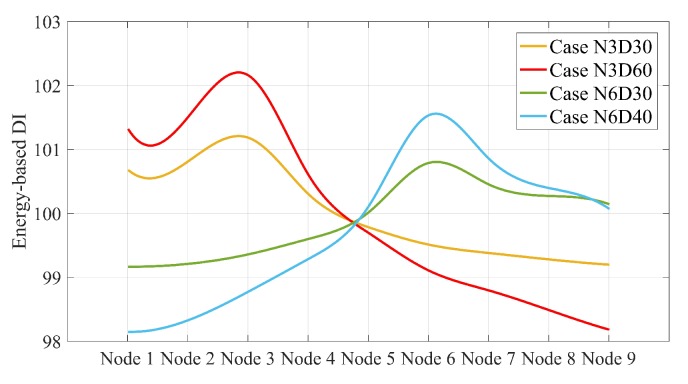
The spline of energy-based DIs along a simply supported beam subjected to moving sprung mass with a speed of 1.25 m/s for all single damage scenarios.

The energy-based DIs are scalar values along the simply supported beam in each scenario. These scalar values can be displayed as the scatter points in the Cartesian coordinate system. Using the spline curve, a smooth line can be determined that connects all the scatter points in each scenario. The splines are shown in Figure 11.

**Figure 12:**
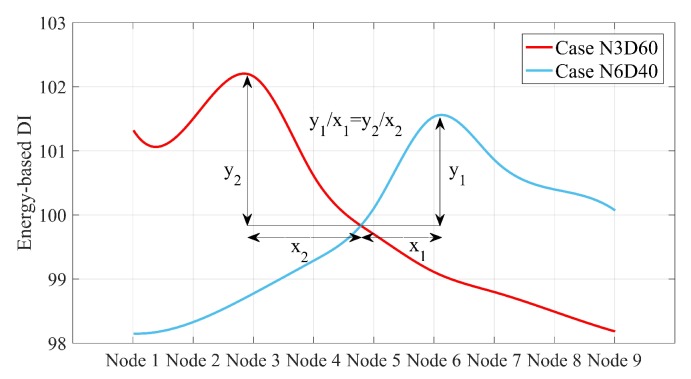
Relationship between maximum splines for cases with the same damage ratio (only for single damage scenarios) under moving sprung mass with a speed of 1.25 m/s.

As shown in Figure 11, the spline of energy-based DIs in each scenario is also maximum at the damage location. However, all these splines have a unique intersection point. Given that the horizontal axis is a normalized axis and each node points to a special distance of the simply supported beam, the intersection point is located exactly at 10.625 m. This intersection can help to quantify the damage by the inverse solution illustrated in Figure 12.

**Figure 13:**
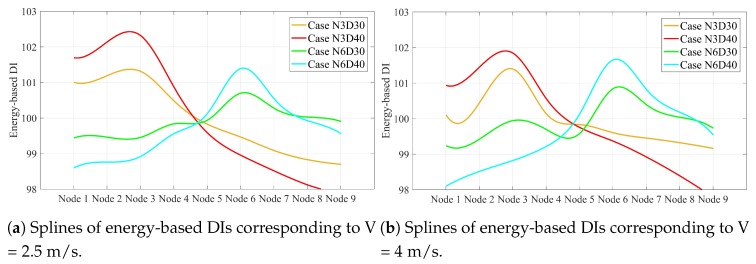
Splines of energy-based DIs along simply supported beam related to velocities (**a**) 2.5 m/s and (**b**) 4 m/s.

Figure 12 proves that the relative gradient of the line between the maximum value of the splines and the intersection of the splines is unique for the scenarios with the same damage scale. Once this slope was calculated for all possible damage scales in a single point on a simply supported bridge, the proposed method could quantify all single damage scenarios along the simply supported beam except the intersection point. It is of note that this slope is not greatly changed by varying the speed. In other words, this slope can be determined using a certain speed, and then used to estimate damage quantification at other speeds. For other velocities considered in this paper, the splines corresponding to the speeds 2.5 and 4 are displayed in Figure 13. Table 5 lists the relative slopes corresponding to different velocities.

Figure 13 shows that the accuracy of damage quantification is decreased by increasing the speed. Table 5 shows that the approximate values of relative slopes are 0.27 and 0.49, which corresponds to 30% to 40% section reduction in the simply supported beam, respectively.

### 4.5. Noise Consideration

**Figure 14:**
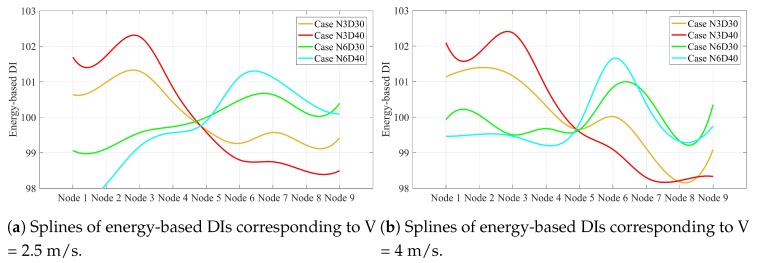
Splines of noisy energy-based DIs along the simply supported beam related to velocities (**a**) 2.5 m/s and (**b**) 4 m/s.

Utilizing experimental data to verify the numerical simulation can help the study to be more reliable because the performance of the proposed model can be evaluated not only by noise-free signals but also under environmental noise. Due to the lack of access to experimental test results in this study, attempts were made to consider the effects of environmental noise manually. In this regard, the recorded acceleration data were polluted with 20% noise. Since the spline of energy-based DIs can locate and quantify the damage, only the spline of energy-based DIs obtained from noisy signals are given in this section. Figure 14 shows the spline of energy-based DI for the noisy acceleration data.

As shown in Figure 14, the splines intersect at 10.625 m from the left side. The splines are able to locate the damage by showing the maximum value along the curve and quantifying the damage by means of the relative slope between the maximum value and the intersection position in the noisy environment. Since both SGF and RDT are noise reduction techniques, the proposed method can accurately locate and quantify the damage. Therefore, it is supposed that the proposed method can essentially reduce the noise during the damage localization process.

### 4.6. Normalization Factor $\mu_{i}$

**Figure 15:**
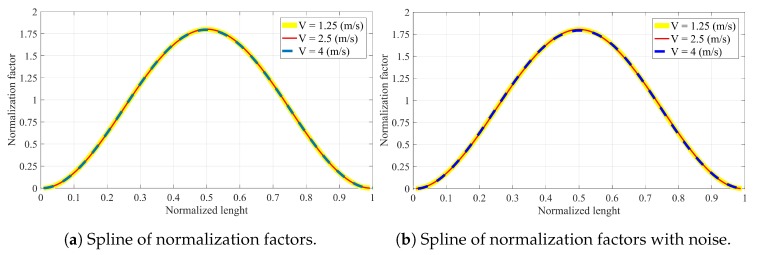
Splines of normalization factors along the non-damaged simply supported beam related to different velocities: (**a**) Without noise and (**b**) with 20% noise.

The normalization factor is a crucial component in the proposed method. Once it is calculated for the non-damage condition, it will serve as the baseline for the damage detection procedure. Table 6 lists the normalization factors for all velocities considered in this paper. The normalization factors of both noisy and noise-free data are connected by the splines (Figure 15). The normalization factors shown in Table 6 are calculated for the noise-free data; however, as shown in Figure 15, the changes imposed by the noise can be ignored.

Figure 15 illustrates three advantages of the proposed method and normalization factor as follows:

1. The spline of normalization factor (SNF) is insensitive to noise (Figure 15a,b are almost the same).
2. The SNF is not changed with the increase of velocity from 1.25 to 4 m/s. Therefore, such insensitivity helps to find the SNF for a certain velocity and then apply it to other velocities. For instance, the SNF for a velocity of 2.5 m/s can be used to find the damage at velocity 1.25 or 4 m/s. This advantage helps to overcome the velocity determination problem and enables the proposed method to accurately work for any velocity between 1.25 to 4 m/s in the process of damage localization and quantification.
3. The SNF follows a Gaussian distribution. The SNF helps to calculate the normalization factors in the locations where no sensor is available. Hence, with SNF, there is no need to install sensors at the same distance before and after the damage.

Regarding sensitivity to noise, location of sensors, and the velocity of the moving load, the proposed energy-based DI is very flexible in dealing with practical issues. Therefore, the proposed method successfully overcomes the practical issues and is able to locate/quantify the damage in bridge-type structures subjected to the moving load.

## 5. Conclusions

This paper proposed a two-stage fully-time-domain damage detection method to locate and quantify the damage in the simply supported beam under the moving sprung mass. In the first stage, the RDT method was used to calculate the RDSs from the acceleration data obtained from a simply supported beam subjected to moving sprung mass. The SGF was then used to calculate the SG-RDSs of all signals in the second stage. The preliminary results showed that the SG-RDSs had only one unique natural frequency, which was the same as the natural frequency of the simply supported beam. A normalization factor and an energy-based DI was proposed to locate and quantify the damage in the simply supported beam. Finally, the proposed method was applied to a numerical case study to localize and quantify the damage while incorporating the noise in the problem formulation.

The main conclusions drawn from this paper are as follows:

- Due to the application of RDT and SGF, the method is not essentially sensitive to noise.
- The method is able to localize and quantify the damage in both noisy and noise-free environments.
- Introducing a general form of baseline by SNF, the proposed method is formulated as a baseline-free method.
- Unlike the conventional methods, the proposed method does not require any sensor at the same distance before and after the damage.
- The proposed method can address multi-damage scenarios in noise-free data.
- The method is flexible in choosing the speed since the SNF is not changed by velocity variations.

Finally, yet importantly, the proposed method locates and quantifies the damage with no need to determine the modal parameters. Moreover, the proposed method does not need prior knowledge of the input excitation and acts as an output-only technique. These two features can significantly reduce the time and cost of calculation.

## Figures and Tables

**Table 1:** Some recommended triggering conditions.

Case NO	Description	Relation
**1**	Level-crossing	$X(t_{i})=a$
**2**	Positive points	$a\leq X(t_{i})<b$
**3**	Zero crossing with a positive or negative slope	$X\left(t_{i}\right)=0,\left\{\begin{array}{c} \dot{X}>0\\ \dot{X}<0 \end{array}\right\}$
**4**	Local extremum	$a\leq X\left(t_{i}\right)<b,\dot{X}=0$

**Table 2:** Properties of the simply supported beam.

Properties	Unit	Symbol	Value
**Length**	m	*L*	25
**Mass per unit**	kg/m	μ	18,360
**Stiffness**	Nm${}^{2}$	EI	4.865 × $10^{6}$

**Table 3:** Properties of the sprung moving mass.

Properties	Unit	Symbol	Value
**Body mass**	kg	$m_{b}$	16,500
**Axle mass**	kg	$m_{t}$	700
**Suspension stiffness**	Nm${}^{-1}$	$K_{s}$	8 × $10^{5}$
**Suspension damping**	Nm${}^{-1}$	$C_{s}$	2 × $10^{4}$
**Tire stiffness**	Nm${}^{-1}$	$K_{t}$	3.5 × $10^{6}$
**Velocity**	m/s	*V*	1.5,2.5,4

**Table 4:** Five damage scenarios considered in the numerical model of the simply supported beam.

Scenario	1	2	3	4	5
**Crack depth to the beam height ratio**	30%	40%	30%	40%	40%
**Location**	At node 3	At node 3	At node 6	At node 6	At node 3 and 6
**Name**	N3D30	N3D40	N6D30	N6D40	N3N6D40

Note: The scenarios are designated with N (damage location) and D (ratio). For example, N6D40 refers to a scenario where the damage ratio is 40% at node 6.

**Table 5:** Slopes corresponding to different velocities and scenarios.

Scenario	Velocity (m/s)	Slope	Scenario	Velocity (m/s)	Slope
N3D30	1.25	0.27	N3D40	1.25	0.49
	2.5	0.30		2.5	0.53
	4	0.32		4	0.42
N6D30	1.25	0.25	N6D40	1.25	0.49
	2.5	0.22		2.5	0.45
	4	0.27		4	0.53
	Average	0.27		Average	0.49

**Table 6:** Slopes corresponding to different velocities and scenarios.

Speed	Node 1	Node 2	Node 3	Node 4	Node 5	Node 6	Node 7	Node 8	Node 9
**1.25**	0.1731	0.6239	1.1783	1.6272	1.7985	1.6270	1.1773	0.6223	0.1725
**2.5**	0.1722	0.6225	1.1789	1.6246	1.8004	1.6275	1.1785	0.6230	0.1724
**4**	0.1725	0.6242	1.1809	1.6283	1.7955	1.6235	1.1772	0.6241	0.1738

## Abbreviations

The following abbreviations are used in this manuscript:

BHM	Bridge health monitoring
BSS	Blind source separation
DI	Damage index
MAF	Moving average filter
RDS	Random decrement signature
RDT	Random decrement technique
SGF	Savitzky–Golay filter
SHM	Structural health monitoring
SG-RDS	Trend line of RDS
SOBI	Second-order blind identification
